# Haploid Production in *Cannabis sativa*: Recent Updates, Prospects, and Perspectives

**DOI:** 10.3390/biology14060701

**Published:** 2025-06-15

**Authors:** S.M. Ahsan, Md. Injamum-Ul-Hoque, Nayan Chandra Howlader, Md. Mezanur Rahman, Md Mahfuzur Rahman, Md Azizul Haque, Hyong Woo Choi

**Affiliations:** 1 Department of Plant Medicals, Andong National University, Andong 36729, Republic of Korea; smvahsan@gmail.com; 2 Department of Agriculture, Gopalganj Science and Technology University, Gopalganj 8100, Bangladesh; mdinjamum92@knu.ac.kr (M.I.-U.-H.); mmahfuz.edu.bd@gmail.com (M.M.R.); 3 Department of Applied Biosciences, Kyungpook National University, Daegu 41566, Republic of Korea; 4 Department of Horticulture, Faculty of Agriculture, Bangladesh Agricultural University, Mymensingh 2202, Bangladesh; nayanhowladar@gmail.com; 5 Institute of Genomics for Crop Abiotic Stress Tolerance, Department of Plant and Soil Science, Texas Tech University, Lubbock, TX 79409, USA; mdmerahm@ttu.edu; 6 Department of Biotechnology, Yeungnam University, Gyeongsan 38541, Republic of Korea; 7 Institute of Cannabis Biotechnology, Andong National University, Andong 36729, Republic of Korea

**Keywords:** *Cannabis sativa*, haploid production, pollen, egg cell, Thiadizuron, CRISPR/Cas9

## Abstract

*Cannabis sativa* L. is a dioecious species known to produce over 1600 chemical constituents, including more than 180 cannabinoids classified into 11 structural groups. These bioactive compounds are predominantly synthesised in the glandular trichomes of female inflorescences. However, sex determination in *C. sativa* is influenced by both genetic and environmental factors, often leading to the development of male flowers on female plants. This unintended fertilisation reduces cannabinoid yield and increases genetic heterogeneity and challenges in medical cannabis production. Haploid and doubled haploid (DH) technologies offer a promising solution by rapidly generating homozygous lines from gametophytic (e.g., unpollinated ovaries and ovules) or sporophytic tissues (e.g., anthers and microspores) via in vitro culture or chromosome reduction during hybridisation. In land plants, the life cycle alternates between a diploid sporophyte and a haploid gametophyte generation, both capable of mitotic division to form multicellular bodies. A single genome regulates this phase transition and encodes the molecular, genetic, and epigenetic mechanisms that precisely control the developmental processes unique to each generation. While the application of haploid technology in *C. sativa* remains limited, through recent progress in haploid induction (HI) and CRISPR-based genome editing, the direct modification of haploid gametes or embryos enables the creation of null homozygous lines following chromosome doubling, improving genetic uniformity. Understanding the molecular mechanisms of spontaneous chromosome doubling may further facilitate the development of elite cannabis genotypes. Ultimately, enhancing the efficiency of DH production and optimising genome editing approaches could significantly increase the speed of genetic improvement and cultivar development in *Cannabis sativa*.

## Simple Summary

*Cannabis sativa*, commonly known as cannabis or hemp, produces many important compounds with medical benefits. However, breeding high-quality cannabis plants is challenging because the species naturally produces both male and female plants, and unwanted pollination reduces the concentration of valuable compounds. One promising solution is a method called haploid culture, which allows scientists and breeders to grow plants from single cells that contain only one set of genetic material. These special plants can then be doubled to create completely uniform, pure lines in just one generation—something that normally takes several years through traditional breeding. In this study, we highlight how haploid culture could help produce stable and consistent cannabis plants more quickly. We also discuss new tools that can work together with haploid culture to make the process more efficient, such as advanced gene editing. While this technology is still developing in cannabis, it offers great promise for improving the speed, precision, and quality of cannabis breeding. This approach could benefit both farmers and patients by making the production of medical cannabis more reliable, controlled, and suited to specific needs.

## 1. Introduction

Industrial hemp (*Cannabis sativa* L.) is a dioecious plant species in the Cannabaceae family [1,2]. *Cannabis* is often associated with its psychoactive compound Δ9-tetrahydrocannabinol (THC), while industrial hemp contains minimal THC content, typically not exceeding 0.3% on a dry weight basis, and mostly synthesises cannabidiol (CBD) [3,4,5,6,7]. Due to its economic relevance, broad adaptability, and potential medicinal value, hemp cultivation is expanding in various regions, particularly across the United States, with the CBD pharmaceutical market emerging as one of the fastest-growing sectors globally.

In addition to THC and CBD, *C. sativa* produces over 100 other phytocannabinoids, accompanied by several terpenoids and flavonoids [3,4,8]. These secondary metabolites (SMs) are synthesised mainly in the glandular trichomes distributed on the floral and foliar tissues of the cannabis plant [3,9]. The cannabinoid biosynthesis pathway initiates with the formation of olivetolic acid (OA) from hexanoyl-CoA through the activity of type III polyketide synthase and olivetolic acid cyclase [3,10,11,12]. OA then condenses with geranyl diphosphate (GPP), derived from the deoxyxylulose phosphate/methylerythritol phosphate (DOXP/MEP) pathway, to form cannabigerolic acid (CBGA) via CBGA synthase [3,10]. CBGA serves as the key precursor for tetrahydrocannabinolic acid (THCA) and cannabidiolic acid (CBDA), catalysed by THCA synthase (THCAS) and CBDA synthase (CBDAS), respectively, which are then non-enzymatically converted into THC and CBD [3,10,13,14]. When divarinic acid is used instead of OA, the pathway shifts toward the production of cannabigerovarinic acid (CBGVA), which can subsequently be converted into cannabidivarinic acid (CBDVA), tetrahydrocannabivarinic acid (THCVA), and cannabichromevarinic acid (CBCVA) [15,16].

**Figure 1:**
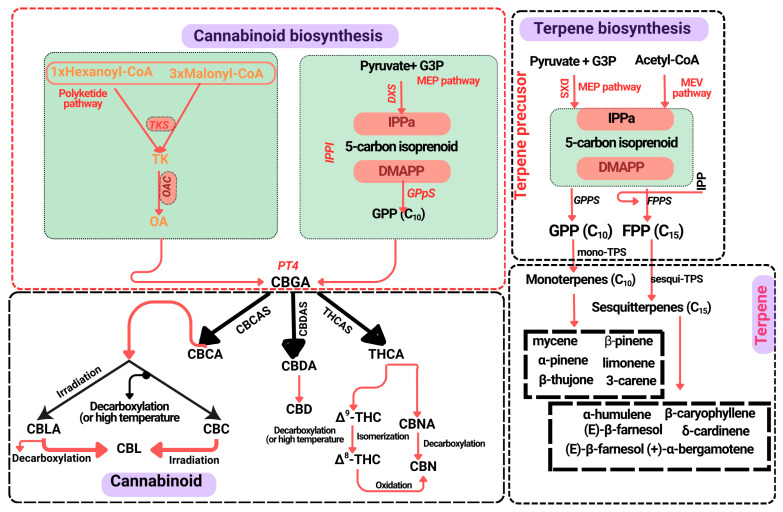
A simplified schematic representation of the cannabinoid and terpene biosynthetic pathways in *Cannabis sativa* L. **Cannabinoid precursors and biosynthesis:** The path begins with olivetolic acid (OA), formed from tetraketide (TK) via tetraketide synthase (TKS) and olivetolic acid cyclase (OAC). Geranyl pyrophosphate (GPP), synthesised by geranyl pyrophosphate synthase (GPPS) from dimethylallyl pyrophosphate (DMAPP) and isopentenyl diphosphate (IPP) through the methylerythritol phosphate (MEP) pathway, is transferred to OA by PT4 (geranylpyrophosphate: olivetolate geranyltransferase 4) to form cannabigerolic acid (CBGA), the central cannabinoid precursor. CBGA is converted into major cannabinoids: cannabidiolic acid (CBDA) via CBDA synthase (CBDAS), tetrahydrocannabinolic acid (THCA) via THCA synthase, and cannabichromenic acid (CBCA) via CBCAS. Following non-enzymatic decarboxylation yields active cannabinoids such as cannabidiol (CBD), Δ^9^-tetrahydrocannabinol (Δ^9^-THC), and cannabichromene (CBC), among others. Minor cannabinoids include cannabinol (CBN), cannabicyclol (CBL), and their acidic precursors (CBNA, CBLA). **Terpene precursors and synthesis:** Terpenes derive from the cytosolic mevalonate (MEV) and plastidial MEP pathways. Farnesyl diphosphate (FPP), synthesised by farnesyl pyrophosphate synthase (FPPS), and GPP, serve as key precursors. Terpene synthase (TPS) enzymes catalyse the formation of diverse mono- and sesqui-terpenes that contribute to the plant’s aroma and therapeutic properties.

Terpene biosynthesis in *C. sativa* operates via two distinct pathways: the plastidial methylerythritol phosphate (MEP) pathway, responsible for monoterpene production, and the cytosolic mevalonate (MEV) pathway, which produces sesquiterpenes [3,17,18]. Enzymes involved in these pathways comprise terpene synthases that catalyse the transformation of GPP and farnesyl diphosphate (FPP) into specific terpene compounds [3,19]. Cannabis flavonoids—primarily flavones and flavonols—occur as aglycones or as O- and C-glycosides. Their biosynthesis is initiated via the phenylpropanoid pathway, where p-coumaroyl-CoA, derived from phenylalanine, combines with malonyl-CoA to produce naringenin, the central precursor for downstream flavonoid synthesis [15,20]. These compounds promote to the plant’s pharmacological profile, with reported applications in treating mood disorders, cancer, diabetes, neurodegenerative conditions, and pain management [1,3,4,21] (Figure 1).

**Figure 2:**
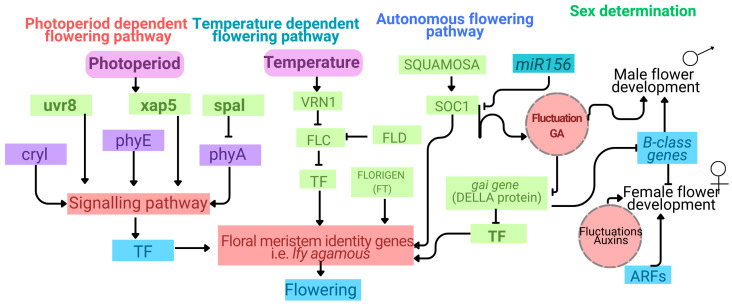
An overview of flowering and sex determination pathways in *Cannabis sativa* regulated by photoperiod, temperature, and endogenous signals, contributing to heterozygosity. The photoperiod pathway includes genes responsible for light perception and signal transduction, such as *uvr8* (UV-B receptor), *xap5* (circadian regulator), *spa1* (suppressor of *PHYA-105*), *cry1* (cryptochrome), *phyA*, and *phyE* (phytochromes A and E). The temperature-responsive flowering pathway involves *vrn1*, a vernalization-associated transcription factor. Both environmental pathways converge to activate endogenous flowering regulators and transcription factors (TFs) that promote the expression of floral meristem identity genes like *lfy* (LEAFY). Key regulators in this endogenous pathway include *FLC*, *FLD*, *FT* (FLOWERING LOCUS T or florigen), *SOC1* (suppressor of *constans1*), and *GAI* (a DELLA protein involved in gibberellin signalling). Additionally, microRNA miR156 contributes to fine-tuning flowering time by modulating gene expression post-transcriptionally. Sex determination is integrated with hormone-regulated metabolic pathways, particularly involving gibberellic acid (GA) and auxin signalling. B-class homeotic genes control male flower development, while female flower differentiation involves auxin response factor (ARF) gene expression. Concurrently, these pathways modulate reproductive organ identity and contribute to the natural dioecy and genetic heterogeneity of *C. sativa*.

Globally, the medical cannabis market has expanded rapidly, valued at USD 3.5 billion in 2019 and projected to reach USD 20.2 billion by 2025. By 2020, more than 50 countries—including China (Yunnan and Heilongjiang), Australia, Germany, Israel, Canada, and much of the United States—had legalised medical cannabis and cannabinoid-based therapies [9,22,23]. *Cannabis* is primarily a diploid and dioecious species, exhibiting a high degree of heterozygosity, as its inheritance does not conform to simple additive or dominant models [23,24]. Although sex chromosomes largely govern floral sex determination, environmental stressors such as photoperiod fluctuation and temperature variation can induce male or hermaphroditic flower formation in female plants, complicating traditional breeding efforts and increasing genetic variability [1,9]. The enzymes THCAS and CBDAS, which compete for CBGA, are key determinants of chemotype. Comparative genome studies have revealed substantial variability in the number of synthase gene copies, though the sequence homology exceeds 90% [23,24]. It is hypothesised that THCAS evolved from CBDAS via gene duplication events, with both genes located in close genomic proximity. Contrary to co-dominant alleles at a single locus, this arrangement has not led to complete THC elimination in CBD-dominant lines [9,24]. Furthermore, the THC: CBD ratio in *Cannabis* genotypes is primarily governed by synthase gene alleles, though environmental and cytogenetic factors—including chromosomal rearrangements, pseudogenes, and maternal inheritance—also influence total cannabinoid levels [24]. de Meijer and colleagues developed a quantitative genetic model to describe cannabinoid content and ratios as complex polygenic traits involving additive and dominant effects [9,23,24]. This underscores the difficulty in stabilising cannabinoid profiles through conventional breeding. The *Cannabis* genome is also highly repetitive and heterozygous, further complicating genetic uniformity and trait fixation [25] (Figure 2).

Haploid plants, occupying a single set of chromosomes derived from either the egg or sperm cell, are classified as maternal or paternal haploids based on their origin. These haploids can go through spontaneous or chemically induced chromosome doubling, producing completely homozygous doubled haploid (DH) lines. In comparison to conventional breeding techniques such as backcrossing and selfing, DH technology swiftly develops genetically stable lines within a single generation [26,27,28]. This methodology significantly lowers breeding timelines and has become a critical tool in advanced plant biotechnology.

Haploid induction occurs early during seed development, facilitated by genes regulating meiosis, fertilisation, gamete interaction, and chromosomal integrity. Enhancements in this area have led to the creation of specialised haploid inducer lines. However, the underlying mechanisms remain complex and often species-specific, limiting the universality of existing haploid systems. The emergence of genome editing platforms, especially CRISPR/Cas9, exhibits new possibilities for inducing haploidy in genetically recalcitrant crops such as *C. sativa*. The direct editing of gametes or haploid embryos can enhance editing efficiency and enable the rapid generation of null homozygous lines following chromosome doubling [26,27,28]. This review synthesises recent advancements in maternal and paternal haploid induction in *Cannabis sativa*, emphasising the genetic, molecular, and technological frameworks essential for establishing robust haploid inducer systems. In particular, it emphasises the application of these tools in breeding programs aimed at improving cannabinoid biosynthesis and accomplishing genetic uniformity in hemp cultivation.

## 2. The Mechanistic and Molecular Background of Haploid and Double Haploid Culture

Haploid induction via androgenesis is commonly achieved by culturing anthers or isolating microspores from anthers. During early development, unicellular microspores or bicellular pollen (1n, haploid nucleus) can deviate from their gametophytic trajectory and initiate embryogenesis [29]. These cells can proliferate into multicellular structures, eventually giving rise to embryos and mature plants with either a haploid or doubled haploid (DH) chromosome number. While androgenesis is a widely utilised approach, gynogenesis—development from female gametophyte cells—offers a valuable alternative, especially in cases where androgenesis is ineffective or incompatible with specific genotypes [30,31]. The female gametophyte, or embryo sac, is embedded within the ovule and attached to maternal tissue via the funiculus. It is located within the nucellus and protected by integuments. A mature embryo sac typically contains four types of cells: the haploid egg cell and two synergids (collectively forming the egg apparatus) at the micropylar end, three antipodal cells at the chalazal end, and a central cell containing two polar nuclei suspended in a large vacuole [32,33,34,35,36,37,38,39]. In dioecious species or male-sterile lines, where pollen-derived haploid induction is not feasible, the induction of haploids from female gametophytic tissues becomes the primary method of choice. Haploid development through gynogenesis can occur in vitro, via ovary or ovule culture, and in vivo, where pollination is unnecessary or limited to triggering development without actual fertilisation [32,33,34,35,36,37,40,41]. Haploid plants can undergo chromosome doubling spontaneously or through induced means (e.g., colchicine treatment), resulting in homozygous DH lines across all loci. Compared to traditional breeding methods like backcrossing and selfing, DH technology allows for the rapid fixation of desired traits within a single generation. These homozygous lines are invaluable for basic genetic studies and applied plant breeding programs [32,33,34,35,36,37,42,43]. DH production is commonly achieved by culturing gametophytic tissues or targeted chromosome elimination during intra- or inter-specific hybridisation [32,44].

Maintaining a diploid (2n) genome is essential for proper vegetative growth and sexual reproduction. Clonal seed development generally requires two main steps: (1) the generation of diploid (2n) pollen and (2) the elimination of either the male or female parental genome post-fertilisation. Usually, male meiosis involves one round of DNA replication followed by two rounds of cell division. Disruptions in early meiotic events—such as homolog pairing, chromosome cohesion, segregation, recombination, spindle organisation, and cytokinesis—can result in the formation of diploid pollen [37,45,46,47,48,49,50]. Notably, the loss of function in specific gene families has been linked to 2n pollen formation [49]. Current in vivo haploid induction (HI) systems can be broadly categorised into three types: (1) CENH3-mediated embryogenesis, (2) haploid induction via parental factors, and (3) transcription factor (TF)-triggered embryogenesis [34,51]. In *Arabidopsis* and maize, functional studies have demonstrated that gamete development and fertilisation abnormalities underlie many of the known HI mechanisms [34,52]. The CENTROMERIC HISTONE H3 (CENH3) protein plays a key role in maintaining diploidy by ensuring accurate chromosome segregation during mitosis and meiosis. While centromere identity is essential for cell division, several structural and regulatory proteins associated with cell cycle progression, chromosomal integrity, DNA replication, and homologous recombination contribute to the haploid–diploid lifecycle [37,45,46,47,48,49,53,54]. Pollen and ovules are under selective pressure to ensure fertilisation and diploid maintenance. However, male gametophytic genes may experience stronger selective forces due to competition among pollen grains. In contrast, female gametes prioritise selective pollen acceptance and resource allocation, reflecting a reproductive strategy focused on optimising fertilisation outcomes [37,45,46,47,48,49,55,56].

**Figure 3:**
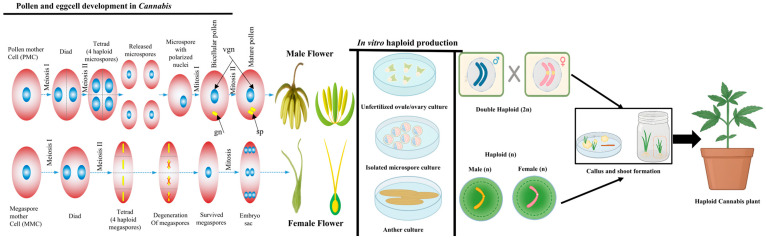
A comprehensive overview of haploid induction in *Cannabis sativa* and other plant species. Pollen development occurs within the anthers of male cannabis flowers, while embryo sac formation follows a monosporic pathway within the ovaries of female flowers. In in vitro haploid induction, explants such as anthers, microspores, or unfertilised ovules are cultured to induce callus formation and adventitious shoots regenerated from these calli may initiate haploid plants. **Abbreviations:** “VGN—vegetative nucleus; GN—generative nucleus; SP—sperm cells.

Compared to in vitro systems, in vivo HI systems offer significant advantages and are further classified into egg cell fertilisation-dependent (eFd-HI) and fertilisation-independent (eFi-HI) systems (Figure 3). In eFd-HI, haploid progeny are generated via pollination with a haploid inducer plant; however, the resulting embryo contains only one parental genome. Although fertilisation of the central cell is still required for viable seed development, the zygotic genome undergoes selective elimination [32,34,45]. Conversely, eFi-HI bypasses pollination entirely. Here, embryogenesis is initiated by ectopically expressing transcription factors, such as BBM (BABY BOOM) or PAR (PARENTHESIS), within the egg cell, thereby activating embryogenic or organogenic pathways independently of gamete fusion [32,34,45,57,58]. Interestingly, the molecular mechanisms driving eFd-HI and eFi-HI appear fundamentally distinct. While eFi-HI relies on TF-driven reprogramming of the egg cell, eFd-HI depends on creating defective sperm or egg cells that simulate fertilisation without enabling the fusion of parental genomes. These defective gametes either fail to contribute their genome to the zygote or are eliminated from the developing embryo [32,34,45,59]. Other pathways implicated in eFd-HI include alterations in centromere functionality, reactive oxygen species (ROS) metabolism, lipid homeostasis, chromosomal damage, and disruptions in gamete or nuclear fusion. Despite significant progress, a comprehensive understanding of in vivo HI remains incomplete. Although mechanistically diverse, the various molecular and cellular processes involved in double fertilisation ultimately converge on key events that dictate haploid generation. Ongoing research aims to integrate synthetic biology and gene engineering approaches to develop programmable HI systems. Such systems would allow the precise control of cellular processes in specific tissues and developmental stages, offering a unified platform for efficient haploid induction across diverse plant species [32,34,45,60,61,62].

## 3. Some Recent Advancements of Haploid Culture in *Cannabis sativa*

Stress treatments are pivotal in inducing microspore embryogenesis, facilitating a shift from the gametophytic to the sporophytic developmental pathway. Physical and chemical stressors—applied to whole plants, inflorescences, flower buds, anthers, or isolated microspores—can effectively trigger this reprogramming, allowing microspores and pollen grains to bypass their standard developmental fate and initiate embryo formation [63,64,65,66]. Among these treatments, cold-shock is the most widely employed method across various species due to its robust ability to induce microspore embryogenesis. The mechanism of action involves cytoskeletal disruption, specifically altering the microtubule organisation unique to microspores and pollen, thereby halting gametophytic division and promoting the onset of sporophytic development [63,67,68]. Cold treatment also activates calcium signalling pathways, elevating intracellular calcium levels and enhancing protein phosphorylation activities associated with cell division and microspore reprogramming [63]. In *C. sativa* cultivars such as USO31 and Finola, developmental stage synchronisation of microspores within the anther has been confirmed through correlation with bud length and cytological analysis. Starch accumulation in these microspores follows a pattern similar to species known to be recalcitrant to androgenesis, suggesting metabolic parallels [63]. Despite low efficiency, the cold-shock pretreatment of buds has been demonstrated to redirect microspores from their gametophytic course to an embryogenic pathway, representing the first documented induction of androgenesis in *C. sativa* and establishing a foundation for doubled haploid research in this species [63]. Additionally, carbohydrate metabolism and physiological regulation within the androecium have supported microspore viability and competence. The presence of amyloplasts, often considered a terminal differentiation marker, further emphasises the importance of carbohydrate dynamics during the transition from gametophytic to sporophytic states. However, because genotypic variation affects responsiveness, the optimisation of pretreatment protocols for each *Cannabis* variety remains essential to achieve consistent and reproducible results [63].

Two primary strategies are used to generate haploid plants: **in situ** and **in vitro** methods. In situ techniques involve special pollination strategies, such as irradiated pollen, interspecific hybridisation, or haploid inducer lines. In contrast, in vitro techniques include the culture of gametophytic cells (e.g., isolated microspores) to regenerate embryos and subsequently haploid plants [69,70]. Microspore culture is generally preferred for its scalability and homogeneity, as millions of cells can be isolated and cultured under controlled conditions. Alternatively, another culture is sometimes used, although it may produce a mix of haploid and diploid plantlets due to somatic cell contribution [46,69,71,72]. One of the most critical factors influencing the success of microspore culture is the developmental stage of the microspore. Only immature microspores—typically at the uninucleate or early binucleate stage—can switch from gametophytic to embryogenic development. This transition occurs near or immediately following the first pollen mitosis, when the microspore remains transcriptionally active and undifferentiated [69,73,74]. Nevertheless, not all cells exhibit embryogenic competence even within a seemingly uniform population of microspores. Minor developmental asynchronies can significantly impact the overall efficiency of the process [69].

**Figure 4:**
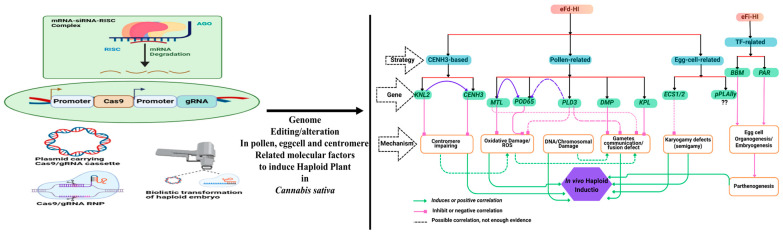
A mechanistic framework of in vivo haploid induction (HI) pathways. HI can occur via egg cell fertilisation-dependent (eFd-HI) or fertilisation-independent (eFi-HI) processes. In eFi-HI, parthenogenesis can be induced by the ectopic expression of genes such as BBM and PAR in the egg cell. In contrast, eFd-HI involves a distinct set of genes that impair molecular processes related to fertilisation and early zygotic development to trigger haploid formation. Haploid Inducer-Mediated Genome Editing (IMGE), or HI-Edit, involves maternal genome editing in crops like maize or wheat. This can be achieved by transient expression of the Cas9/gRNA cassette or siRNA during HI. Isolated microspores can also be edited via transfection with either DNA-based Cas9/gRNA constructs or ribonucleoprotein (RNP) complexes, followed by the regeneration of edited haploids. Additionally, direct editing of haploid tissues can be performed using biolistic delivery systems carrying transgenes or Cas9/gRNA RNPs. Abbreviations: BBM—BABY BOOM; CENH3—centromeric histone H3; KNL2—KINETOCHORE NULL 2; MTL—MATRILINEAL; POD65—PE-ROXIDASE 65; PAR—PARTHENOGENESIS; PLD3—PHOSPHOLIPASE D3; ECS1/2—EGG CELL-SPECIFIC ASPARTIC ENDOPEPTIDASE 1/2; DMP—DOMAIN OF UNKNOWN FUNCTION 679; KPL—KOKOPELLI; pPLAIIγ—GYNOECIUM-EXPRESSED PHOSPHOLIPASE AII; EC1—EGG CELL 1; HAP2—HAPLESS 2; GCS1—GENERATIVE CELL SPECIFIC 1”.

In the broader context of plant biotechnology, efficient regeneration systems are essential for the successful implementation of genetic transformation methods, particularly those with inherently low efficiency, such as *Agrobacterium*-mediated transformation and CRISPR/Cas-based gene editing [22,75,76,77]. High-regeneration culture systems provide an ideal foundation for these applications. Haploid microspores, in particular, offer a desirable target for genome editing due to their single set of chromosomes. Edits introduced at the haploid stage can be stably fixed through chromosome doubling, resulting in homozygous mutant lines without chimerism or the need for successive inbreeding generations. This significantly reduces the time and cost required for generating stable transgenic or gene-edited lines [33,75,78]. The assimilation of haploid induction and CRISPR-based editing presents a transformative tactic for the genetic improvement of *C. sativa*. It hastens cultivar development and facilitates the precise manipulation of traits such as cannabinoid content, flowering behaviour, and stress tolerance. By combining advanced microspore culture systems with efficient gene editing platforms, breeders and researchers can create genetically uniform, high-performing starting materials that streamline F1 hybrid development and meet the rigorous demands of both the industrial and pharmaceutical cannabis areas [67,75,79] (Figure 3 and Figure 4).

## 4. Prospects of RNAi and CRISPR/Cas-Mediated Tools for Genome Editing Towards Haploid *Cannabis sativa* Development and Secondary Metabolism Biosynthesis

Recent advancements in genetic engineering and genome editing have significantly enhanced the potential of modern agriculture, particularly when combined with reliable systems for the propagation and regeneration of genetically modified or edited plants. One of the most notable advantages of androgenesis is its capacity to rapidly fix homozygosity, making it highly valuable in breeding programs. Doubled haploid (DH) cultivars are now cultivated globally, as their completely homozygous genetic makeup makes them ideal for constructing molecular maps, dissecting quantitative trait loci (QTLs), conducting marker-assisted selection (MAS), facilitating mutation screening, performing reverse breeding (RB), and implementing genome-wide association studies (GWAS). These homozygous lines also serve as essential platforms for validating gene function through strategies such as targeting induced local lesions in genomes. Currently, two main in vitro breeding (IVB) approaches are employed for haploid induction: androgenesis and induced parthenogenesis. The latter involves rescuing parthenogenic embryos generated via pollination with irradiated pollen or through the direct culture of ovaries and ovules [34,37,45,48]. While female reproductive structures are the source in parthenogenesis, haploid induction in androgenesis originates from male gametophytic cells. The foundational methodologies for IVB are further outlined in [80]. Mutation breeding, another widely utilised strategy, involves the application of physical or chemical agents to generate heritable genetic changes. Although spontaneous mutations occur at a very low frequency (approximately 10^−6^), mutagenesis using physical factors such as X-rays, gamma rays, or cosmic rays, and chemical agents like EMS, EES, or EI, significantly enhances the likelihood of obtaining desirable genetic variants [81]. Such techniques, whether applied in vivo or in vitro, effectively broaden the genetic base and improve key agronomic traits at a higher frequency than spontaneous mutations. Recent studies also explore the phenomenon of albinism observed during anther culture from an epigenetic perspective. Haploid plants, which carry a single set of chromosomes, exemplify cellular totipotency and serve as a bridge for generating fully homozygous lines through chromosome doubling. DH technology is instrumental in both developmental biology and applied breeding [37,45,48]. These homozygous plants are also amenable to stable genetic transformation using various delivery systems such as electroporation, biolistics, in planta particle bombardment (iPB), and *Agrobacterium*-mediated transformation [37,45,48]. Gene transformation via isolated microspore culture can proceed through gametophytic or sporophytic pathways. In the gametophytic approach, foreign DNA is introduced into mature pollen, stigmas, or microspores, followed by pollination with the transformed material—a strategy known as male germline transformation. Alternatively, in the sporophytic pathway, embryogenic microspores are used as explants, and genome doubling of transformed haploids results in homozygous transgenic lines. The use of modern genome editing platforms—including TALENs, ODM, ZFNs, and especially CRISPR/Cas—has shown great promise in generating DH lines with precise, desirable traits within a single generation [37,45,48]. Genome editing has rapidly emerged as a focal area in plant research [82,83]. Among the diverse tools available, the CRISPR/Cas system stands out for its specificity, efficiency, and modularity. Variants such as dCas9 (catalytically inactive Cas9) retain a DNA-binding capacity but lack cleavage activity, allowing for the manipulation of gene expression via knockouts, knock-ins, base editing, and gene activation or repression [82,83]. When fused with transcriptional activators, dCas9 enables a fine-tuned regulation of key transcription factors such as AP2/ERF, WRKY, bHLH, bZIP, MYB, and NAC, all of which are central to secondary metabolite biosynthesis in *Cannabis sativa* [84,85,86]. CRISPR activation (CRISPRa) techniques leverage this system to enhance transcriptional activity by targeting specific promoter regions [82,83].

Additionally, CRISPR/Cas-induced double-strand breaks (DSBs), guided by sequence-specific sgRNAs, activate endogenous DNA repair mechanisms—namely, homologous recombination (HDR) or non-homologous end joining (NHEJ)—which can be exploited for gene insertion, deletion, or regulatory fine-tuning. CRISPR interference (CRISPRi), based on dCas9, provides a robust alternative to RNAi for transcriptional silencing in both prokaryotic and eukaryotic systems [87]. By blocking RNA polymerase progression at specific genomic loci, this approach offers a transient yet effective method for gene suppression—ideal for manipulating metabolism or developmental pathways during haploid induction in *Cannabis sativa* [82,87].

Furthermore, epigenetic modifiers such as DNA demethylases and histone deacetylase inhibitors are increasingly being used in combination with in vitro techniques to drive targeted genetic and epigenetic changes [80,81,88]. CRISPR/Cas9-based editing, when integrated with *Agrobacterium*-mediated transformation and plant tissue culture systems, enables the precise genetic manipulation of complex traits within a considerably shorter breeding cycle compared to traditional methods [80,81,88]. Epigenetic mechanisms—including DNA methylation, histone modification, and chromatin remodelling—govern the expression of secondary metabolite biosynthetic genes, which are often silenced in heterochromatin regions [82,89]. Small-molecule epigenetic regulators that modulate chromatin accessibility are thus promising tools for activating silent gene clusters and enhancing the production of valuable metabolites [82,89]. With the flexibility offered by dCas-based epigenetic effectors, researchers can now precisely control the chromatin landscape, gene expression patterns, and developmental processes in *Cannabis sativa* [82,89,90].

When messenger RNA (mRNA) enters the RNA interference (RNAi) pathway, it is cleaved into small interfering RNAs (siRNAs), which then guide the degradation of complementary mRNA targets [91]. This results in mRNA degradation, post-transcriptional silencing, and occasionally, transcriptional silencing [92,93]. Recently, RNAi has been optimised to silence specific genes involved in cannabinoid biosynthesis in *Cannabis sativa* [3,10,23]. Both RNAi and CRISPR/Cas9 are used for gene silencing, but operate via different mechanisms. CRISPR/Cas9 is a more advanced technology, though it is still developing. In contrast, RNAi is well established, with more extensive protocols and available libraries, making it easier to apply [92,93]. Notably, phenotypes induced by non-transgenic RNAi methods are not heritable, which facilitates broader applications in genetic research. Each approach has specific advantages and limitations, and their selection depends on research objectives [92,93]. Tissue culture-independent methods, such as spray-induced gene silencing (SIGS), virus-induced gene silencing (VIGS), or virus-induced gene editing, offer promising tools for accelerating functional genomics studies [94]. Moreover, emerging techniques like base and prime editing, orthogonal synthetic transcription factors, and synthetic directed evolution present new possibilities for precise trait improvement [94].

The use of RNAi to induce haploid lines in onion through genome elimination, targeting the centromeric variant of histone 3 (CENH3), resulted in poor seed set and segregation distortion, and no homozygous knockdown lines were recovered, highlighting the limitations despite the relevance of RNAi-induced doubled haploids in breeding programmes [95]. Nonetheless, this approach holds promise for application in crop species where CRISPR/Cas9-based gene knockout is not viable [95]. A key gene in *planta* haploid induction is a pollen-specific phospholipase A, which, when mutated, has been shown to induce haploids in several monocot species. However, no functional ortholog gene has been identified in dicot plants to date [96]. An RNAi-mediated loss-of-function study in Arabidopsis targeting the gynoecium-expressed phospholipase AII (pPLAIIγ) resulted in maternal haploid induction at an average frequency of 1.07% [96].

While RNA interference (RNAi) and CRISPR/Cas9 were historically the primary methods for suppressing gene expression, both present limitations—RNAi is cytoplasm-restricted and requires nearly perfect mRNA complementarity, while CRISPR/Cas9 can exhibit off-target activity and cytotoxicity [82,97]. CRISPR-Cas13a offers an RNA-targeted alternative, functioning via HEPN-domain RNase activity to regulate gene expression at the transcript level [82,98].

This system provides an efficient, non-permanent method to edit or degrade RNA in vivo, enabling novel insights into post-transcriptional gene regulation. Base editors—such as cytidine base editors (CBEs) and adenine base editors (ABEs)—have further expanded the CRISPR toolkit by allowing single-nucleotide changes without DSBs or donor templates [82,98,99]. Although structural variation editing using Cas9 and paired sgRNAs is still in its infancy in *Cannabis sativa*, this approach holds potential for studying genome architecture and regulatory networks. Meanwhile, the CRISPR-Cas12a system has gained momentum for plant genome editing due to its compatibility with T-rich PAM sequences and its ability to produce staggered cuts that enhance gene integration efficiency [82,83,100,101]. Cas12a also supports multiplexing through a single CRISPR array and is particularly well suited for targeting AT-rich regions like promoters and introns. Though it was once limited by temperature sensitivity, newly engineered Cas12a variants have overcome this barrier, enhancing its utility in plant systems [82,83,100,101].

Ultimately, the convergence of DH technology with CRISPR genome editing enables transformative breeding strategies. For instance, pollination with CRISPR–Cas9-expressing haploid inducer lines has produced fully edited, inbred plants in just two generations [102]. Moreover, multiplex genome editing with gRNA arrays allows the simultaneous modification of ten or more loci, accelerating trait pyramiding. Compared to backcrossing, combining DH and CRISPR technologies can improve breeding efficiency by at least tenfold and offers the additional advantage of producing transgene-free edited lines by eliminating the male donor genome during haploid induction, alleviating public concerns regarding genetically modified organisms [102].

## 5. The Morphoregulatory Role of Thidiazuron In Vitro Regeneration of *Cannabis sativa*: An Unexplored Potential for Haploid Production in *Cannabis sativa*

Thidiazuron (TDZ), a synthetic diphenylurea compound, functions both as a herbicide and a potent plant growth regulator [103]. It is commonly applied to defoliate cotton plants and has proven particularly useful in promoting regeneration in recalcitrant species under in vitro conditions [104,105]. What sets TDZ apart is its unique ability to mimic the physiological actions of auxins and cytokinins, despite being structurally distinct from both. It has been suggested that TDZ triggers the activation of certain genes and regulatory elements associated with callus induction, either directly or by stimulating endogenous auxin or cytokinin biosynthesis [106,107]. A hallmark of cytokinin function is its promotion of plant cell division, especially during the G1/S and G2/M phases of the cell cycle, which recent studies have increasingly linked to cytokinin signalling pathways [106,108]. TDZ has been shown to effectively support cytokinin-dependent callus development, a property widely utilised in horticultural propagation [109,110]. Upon metabolism, TDZ is cleaved at its amide linkage, releasing biologically active metabolites, many of which contain organic nitrogen and sulphur, that influence plant tissue responses [106]. Metabolomic studies have proposed six primary hypotheses regarding TDZ function, including an enhanced sugar uptake, elevated primary metabolic activity, rerouting of terpene pathways, and modulation of stress responses through indoleamine and phenylpropanoid metabolism [106,108]. Although TDZ is often described as exhibiting adenine-type cytokinin-like activity—either by promoting the synthesis of endogenous cytokinins or binding to cytokinin receptors—this model does not fully explain the range of physiological effects observed across plant species [106,108]. In certain systems, TDZ elicits responses more akin to exogenous auxins, and some evidence suggests that it enhances endogenous auxin biosynthesis. Overall, the physiological impact of TDZ appears to involve a sophisticated interplay among multiple hormone pathways, or phytohormone crosstalk, influencing morphogenetic outcomes. Additionally, the effects of TDZ are strongly influenced by dosage, exposure duration, light conditions, and other environmental factors. Despite its long-standing use and commercial significance, the exact mechanism(s) by which TDZ regulates plant development remain elusive [106,108].

TDZ has also demonstrated effectiveness in haploid induction due to its capacity to promote adventitious shoot or callus formation from haploid-derived explants [106,111,112,113,114]. Its role in facilitating chromosomal reduction is especially critical in the development of haploid plants. In ornamental species, TDZ has been successfully employed to induce embryogenesis from microspores during the androgenesis process [106,111,112,113,114]. When combined with other promotive conditions, TDZ enhances doubled haploid (DH) production protocols, affirming its relevance and potential utility in breeding strategies [36,115,116,117,118]. Though TDZ has recently been applied in *Cannabis sativa* regeneration from various explant types [119,120], its application in haploid culture systems remains largely unexplored. The success of TDZ-induced callogenesis or shoot organogenesis depends on several key factors: (1) the concentration used in the culture medium; (2) the duration of explant exposure; (3) whether it was used independently or in combination with other growth regulators; (4) the nature of any co-applied compounds; and (5) the specific plant species in question [46,106,111].

## 6. Conclusions

Haploid plants, as sporophytes carrying gametophytic chromosome numbers, exemplify cellular totipotency and represent powerful systems in both fundamental and applied plant science. Derived from gametophytic cells, they contain only half the chromosome set of somatic or zygotic cells and serve as critical materials in genetics, crop improvement, and developmental biology. Doubled haploid (DH) lines, being completely homozygous, are particularly well suited for stable gene transformation using various delivery platforms such as electroporation, microprojectile bombardment, *in planta* particle bombardment, and *Agrobacterium*-mediated transformation. Gene transfer approaches through isolated haploid cultures include gametophytic and sporophytic pathways, such as CENH3-based systems and fertilisation-dependent or independent HI (eFd-HI and eFi-HI). Male germline transformation through DNA delivery into microspores or mature pollen exemplifies the gametophytic route, whereas embryogenic microspores used in transformation represent the sporophytic approach, with subsequent chromosome doubling resulting in homozygous transgenic plants. Modern genome editing systems—particularly CRISPR/Cas9—enable the highly efficient production of DH lines with tailored traits in a single season. The integration of artificial intelligence into this field further enhances the precision of gene function prediction and accelerates genome editing, supporting rapid development of elite cultivars. The conjunction of in vitro regeneration systems with gene editing tools and computational modelling holds great promise for the long-term conservation, improvement, and sustainable exploitation of *C. sativa*. DH populations, due to their genetic uniformity, are instrumental in quantitative trait loci (QTL) mapping, marker-assisted selection (MAS), mutation screening, reverse breeding (RB), and genome-wide association studies (GWAS). These systems also provide the finest platforms for validating gene function. In addition, the success of haploid-based regeneration depends profoundly on the choice of culture media and plant growth regulators (PGRs), particularly in pollen and ovule germination. Transcription factors (TFs) are central regulators of indirect embryogenesis, controlling gene expression networks that mediate embryogenic cell formation and differentiation. These TFs serve as master switches in transitioning somatic cells into embryogenic states. Moreover, epigenetic modifications such as DNA methylation and chromatin remodelling play essential roles in reprogramming the epigenome, which is important for callus induction and morphogenic competence. Understanding these layers of transcriptional and epigenetic regulation is key to improving regeneration protocols, specifically in recalcitrant species like *Cannabis sativa*. Future studies integrating a CRISPR-based functional validation of candidate genes will be dynamic for deciphering the molecular framework controlling callus formation and shoot regeneration from haploid tissues, thereby overcoming current bottlenecks in cannabis biotechnology and breeding.

## Data Availability

Not Applicable.
